# Effect of Growth Temperature and Atmosphere Exposure Time on Impurity Incorporation in Sputtered Mg, Al, and Ca Thin Films

**DOI:** 10.3390/ma16010414

**Published:** 2023-01-01

**Authors:** Shamsa Aliramaji, Philipp Keuter, Deborah Neuß, Marcus Hans, Daniel Primetzhofer, Diederik Depla, Jochen M. Schneider

**Affiliations:** 1 Materials Chemistry, RWTH Aachen University, Kopernikusstr. 10, D-52074 Aachen, Germany; 2 Department of Physics and Astronomy, Uppsala University, Box 516, S-75120 Uppsala, Sweden; 3 Department of Solid State Sciences, Ghent University, Krijgslaan 281 (S1), B-9000 Gent, Belgium

**Keywords:** impurity incorporation, physical vapor deposition (PVD), thin film

## Abstract

Impurities can be incorporated during thin film deposition, but also can originate from atmosphere exposure. As impurities can strongly affect the composition—structure—property relations in magnetron sputter deposited thin films, it is important to distinguish between both incorporation channels. Therefore, the impurity incorporation by atmosphere exposure into sputtered Mg, Al, and Ca thin films is systematically studied by a variation of the deposition temperatures and atmosphere exposure times. Deposition temperature variation results in morphological modifications explained by considering surface and bulk diffusion as well as grain boundary motion and evaporation. The film morphologies exhibiting the lowest oxygen concentrations, as measured by energy dispersive X-ray spectroscopy, are obtained at a homologous temperature of 0.4 for both Mg and Al thin films. For Ca, preventing atmosphere exposure is essential to hinder impurity incorporation: By comparing the impurity concentration in Al-capped and uncapped thin films, it is demonstrated that Ca thin films are locally protected by Al-capping, while Mg (and Al) form native passivation layers. Furthermore, it can be learned that the capping (or self-passivation) efficiency in terms of hindering further oxidation of the films in atmosphere is strongly dependent on the underlying morphology, which in turn is defined by the growth temperature.

## 1. Introduction

Impurity incorporation and therefore variation in chemical composition results in modification of interatomic bonding [1], thin film microstructure [2], and consequently also the properties [3,4]. Furthermore, oxygen diffusion was reported along crystal defects where structural changes were induced in the presence of oxygen at these defects [5]. Therefore, controlling impurities is crucial for adjusting properties [6,7,8,9,10]. Impurity incorporation occurs either during deposition (growth-related impurity incorporation), or after film synthesis, when the film is exposed to atmosphere. Factors that affect the impurity incorporation by atmosphere exposure include exposure time, venting temperature, and film morphology [11,12,13].

Venting temperature determines surface composition and therefore impurity incorporation of thin films. An extensive study has been carried out on TiN films in this regard [12]. It has been demonstrated that the surface composition is strongly dependent on venting temperature [12]. Therefore, the venting temperature should be as low as possible to minimize surface composition modifications due to atmosphere exposure during venting.

However, as soon as the as-deposited film is taken out of the vacuum system, it is exposed to the atmosphere which contains reactive gases such as O_2_, CO_2_, H_2_O, and H_2_ [14]. Therefore, these species can potentially be incorporated into the films depending on the reactivity of the thin film material [15]. For example a continuous increase in lattice parameter upon atmosphere exposure was reported for HfV_2_ thin films due to interstitial O incorporation [13,16]. One of the possibilities to avoid such impurity incorporations is using a thin protective capping layer [17,18,19]. Protection of HfV_2_ from O incorporation was achieved by an approximately 10 nm thick Nb capping layer [13].

It is well known that the synthesis temperature affects thin film morphology, surface roughness, and also film density which in turn can play an important role in the impurity incorporation by atmosphere exposure because the topography as well as the film density determine the surface area in contact with the atmosphere [2,20,21]. For low homologous temperatures, thin film morphology evolution may be determined by surface diffusion. In the absence of surface diffusion, atomic shadowing results in the formation of voided grain boundaries and hence porosity, enabling efficient impurity incorporation via diffusion along the voided grain boundaries [22,23].

In the present work, impurity incorporation through atmosphere exposure is systematically studied for Mg, Al, and Ca thin films deposited at room temperature and for substrate temperatures of up to 300 °C. The corresponding morphological variations can be explained by considering synthesis temperature-induced activation of mobility at the surface in grain boundaries as well as in the bulk.

## 2. Materials and Methods

Thin film deposition was carried out by direct current magnetron sputtering in a high vacuum chamber at a target to substrate distance of 10 cm using 50 mm diameter elemental targets of Mg (99.95%), Al (99.99%), and Ca (99.5%) on 50.8 ± 0.3 mm diameter Si (100) substrates at floating potential. For the synthesis of all thin films, the substrate was rotated at 15–20 rpm. The deposition chamber was evacuated to a base pressure below 8 × 10^−6^ Pa measured at room temperature prior to the start of the deposition by an IONIVAC Transmitter ITR 200 S pressure gauge containing a hot cathode ionization Bayard–Alpert gauge combined with a Pirani sensor.

As the Ca target was received in mineral oil, prior to installation the oil on the target surface was removed by multiple immersions into n-hexane (98.5%) in a glovebox continuously flushed with Ar (99.999%) where the cleaned Ca target was then mounted on the cathode. The cathode was subsequently transferred to the deposition chamber in ambient air where atmosphere exposure could not be avoided.

For Ca, extensive sputter cleaning was carried out (up to 360 min) prior to deposition with pulsed-DC sputter cleaning at a frequency of 250 kHz and duty cycle of 60% at a minimum applied power of 20 W and continuously increasing the power up to 200 W following the same gradual increase in applied power from 20 to 200 W procedure under DC mode. The Mg and Al targets required a cleaning time of approx. 30 min at 200 W and could be sputtered without arcing after conditioning the target, however, it should be noted that in case of Ca, an average of 4 arcs/min was observed during deposition. Thin films with a thickness of approximately 1 μm were deposited using Ar with a purity of >99.999% as sputtering gas to achieve a working pressure of 0.5 Pa with a flow rate of 28.3 sccm. The substrate temperature was varied from room temperature (without intentional heating, denoted as RT where the maximum measured temperature was ≤50 °C) up to 300 °C which leads to an increase in base pressure. The Al-capping layer with a thickness of approximately 70 nm was deposited for 3 min at a substrate temperature of ≤50 °C at 200 W. Details of the synthesis parameters are summarized in Table 1, where the variation in base pressure is caused by the “history” in terms of previously sputtered material, previous substrate temperature, previous base pressure, time available for pumping, etc. The ratio between the material flux, calculated from deposition rate of the growing films, and the calculated impurity flux is ≥69 for all deposition experiments reported here.

Chemical composition analysis was performed by energy dispersive X-ray spectroscopy (EDX, EDAX Inc., Mahwah, NJ, USA) using an EDAX Genesis 2000 analyzer in a JEOL JSM 6480 scanning electron microscope (SEM, JEOL Ltd., Tokyo, Japan). The acceleration voltage was set to 8 kV and the measurement time was 100 s at a magnification of 1000×. Background deconvolution was optimized with respect to the O Kα transition. Error bars correspond to standard deviations determined from 3 to 5 EDX measurements. Chemical composition depth profiling was carried out by time-of-flight elastic recoil detection analysis (ToF-ERDA, non-commercial instrument) at the Tandem Laboratory of Uppsala University. Then, 36 MeV ^127^I^8+^ primary ions were directed onto the thin films at an incidence and exit angle of 22.5°. The pressure during analysis was < 2× 10^−6^ mbar. A time-of-flight telescope with thin C foils was used [24] and the time and energy coincidence spectra were analyzed with the CONTES software package [25]. The systematic uncertainty of 10% relative of the deduced values for O and H originates from uncertainties of stopping powers from primary ions as well as the recoiling particles as discussed in the supplementary material of to Baben et al. [26]. Conversion of the areal density of depth profiles to nanometers was accomplished using a calculated density of 1.7 and 2.7 g cm^−3^ for Mg and Al, respectively [27,28]. Error bars correspond to the standard error of the measured concentrations in depth profiles of thin films excluding the 100 nm region adjacent to the surface.

Structural analysis of the films was performed using a Bruker D8 General Area Detection Diffraction System (GADDS, Bruker Corporation, Billerica, MA, USA) with Cu Kα radiation. The voltage and current settings were 40 kV and 40 mA, respectively. The angle of incidence was kept fixed at 15° whereas the 2θ range was 15° to 75°. Selected samples were analyzed by XRD using Bragg–Brentano geometry in Siemens D5000 X-ray Diffraction System (Siemens AG, Munich, Germany). Coupled (2° shift) Bragg–Brentano scans with an increasing 2θ from 10° to 90° were used. Microstructural characterization was carried out on thin lamellae which were prepared in a FEI Helios Nanolab 660 dual-beam microscope (Thermo Fisher Scientific, Waltham, MA, USA) by focused ion beam (FIB) techniques. The platform is also equipped with a STEM III detector as well as an Octane Elect EDX device (EDAX Inc., Mahwah, NJ, USA). Micrographs of thin film cross-sections were acquired in bright-field (BF) mode at an acceleration voltage of 30 kV and a current of 50 pA. The surface topography was also studied with a Keyence VK-9700 laser optical microscope (LOM, Keyence, Osaka, Japan).

## 3. Results and Discussions

Generally, impurity incorporation in thin films occurs either during deposition (growth-related impurity incorporation), or after synthesis, upon atmosphere exposure. The latter impurity incorporation pathway must be avoided to study the former. In an effort to avoid surface composition modification upon atmosphere exposure, Al-layers with a thickness ranging from 1.5 to 6 nm were employed as these were shown to serve as effective barriers to hinder oxidation of the underlying film [17,18,19]. Therefore, initially, the functionality of such barrier capping layers was investigated for the thin film materials of interest.

### 3.1. Al Capping to Prevent Impurity Incorporation by Atmosphere Exposure

To investigate the Al-capping functionality, around 70 nm thick, Al-capping layer was deposited onto the as-deposited films after cooling down from the synthesis temperature to approx. ≤ 50 °C in an effort to avoid interdiffusion of Al into the underlying film prior to venting and subsequent atmosphere exposure [29].

**Figure 1:**
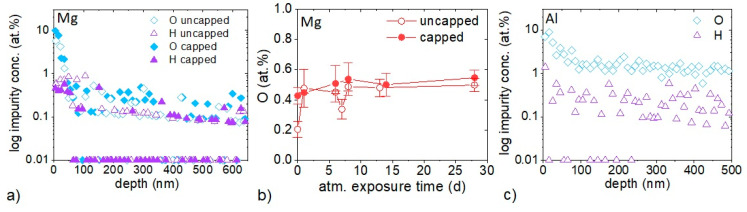
Impurity uptake in Al-capped and uncapped Mg, as well as in uncapped Al films all deposited at 100 °C: (**a**) ERDA composition depth profiles of O and H impurities after 86 days of atmosphere exposure of uncapped and capped Mg. (**b**) EDX measured O concentration as a function of their atmosphere exposure time (expressed in days (d)) after synthesis for capped and uncapped Mg films. (**c**) ERDA composition depth profiles of O and H impurities after 87 days of atmosphere exposure into uncapped Al. Al-capping layers were deposited at ≤50 °C.

The Al-capping functionality is investigated for Mg films deposited at 100 °C. ERDA depth-resolved chemical composition analysis was performed after 86 days of atmosphere exposure for uncapped and Al-capped Mg as well as uncapped Al films deposited at 100 °C (Figure 1a,c). Furthermore, repeated EDX measurements were done after atmosphere exposures of up to 28 days for Al-capped and uncapped Mg films, Figure 1b.

ERDA depth profiles of both Al-capped and uncapped Mg films after 86 days of atmosphere exposure demonstrate a higher surface O concentration for the capped film compared to that of the uncapped film where the majority of the O originates from the surface, whereas similar impurity concentrations are obtained by neglecting 100 nm of the surface (Figure 1a). The O concentrations calculated in a depth ranging from 100 to 650 nm based on ERDA, are 0.2 ± 0.1 and 0.1 ± 0.1 at % and H concentrations are 0.1 ± 0.1 and 0.1 ± 0.1 at % for capped and uncapped Mg films, respectively. Furthermore, irrespective of synthesis conditions, a C concentration of 0.2 ± 0.1 at % is observed in all the deposited films of this study based on ERDA (neglecting 100 nm of the surface). Moreover, comparing the impurity incorporation upon continuous atmosphere exposure of capped and uncapped Mg films deposited at 100 °C exhibits very similar and largely exposure time-independent O concentration range of 0.2 ± 0.1 to 0.5 ± 0.1 at % based on EDX (Figure 1b). In the EDX data, contrary to the ERDA data, the surface reaction layer formed due to atmosphere exposure, contributes to the reported oxygen concentration. Thus, no influence of Al-capping is observed for the impurity incorporation of the here-studied Mg films (neglecting the surface reaction layer).

Furthermore, the depth profile of uncapped Al film after 87 days of atmosphere exposure (Figure 1c) demonstrates O and H uptakes averaged over a depth range of 100 to 500 nm of 1.4 ± 0.1 and 0.2 ± 0.1 at %, respectively and hence, significantly higher impurity concentrations than measured for the uncapped Mg, see Figure 1a. Therefore, Mg thin films deposited at 100 °C, see Figure 1a, provide a more efficient surface passivation than Al thin films deposited at 100 °C, see Figure 1c.

**Figure 2:**
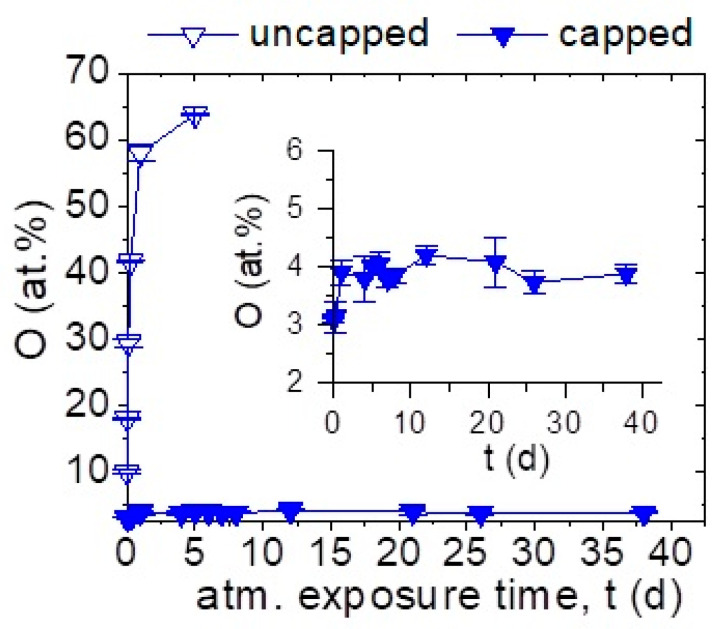
EDX measured O concentration in Al-capped and uncapped Ca thin films deposited at 100 °C as a function of their atmosphere exposure time after synthesis, expressed in days (d). Delamination occurs after 5 days of atmosphere exposure preventing further measurement of uncapped Ca. The inset shows detailed information of O concentration of capped Ca film. Al-capping layers were deposited at ≤50 °C.

The Al-capping functionality is further investigated for elemental Ca films deposited at 100 °C by measuring the local O concentration repeatedly by EDX after atmosphere exposure of up to 38 days, see Figure 2. Measurements were conducted in regions with intact capping, which will be discussed in more detail below.

In the as-deposited state, the Al-capped Ca film exhibits an averaged O concentration of 3.1 ± 0.1 at % whereas a concentration of 10.0 ± 0.4 at % was measured for the uncapped Ca after ≤3 min atmosphere exposure during sample transfer between synthesis and analysis experiments. Comparison of the O concentrations in the two films in the as-deposited state indicates that most of the O in the uncapped Ca film is incorporated during atmosphere exposure. Moreover, uncapped Ca shows a continuous O uptake upon atmosphere exposure. This is in agreement with studies on bulk Ca where a high reactivity with water vapor was observed [30,31]. After 5 days, the O concentration in uncapped Ca was measured to be 63.8 ± 0.1 at % approaching the nominal chemical composition of CaO_2_ or more likely Ca(OH)_2_ (EDX is not sensitive to H) as the peroxide is not stable upon atmospheric exposure. Thereafter, the film delaminated preventing further measurements. In contrast to the pronounced O uptake in uncapped Ca, the O concentration of the capped film is measured to be constant indicating that the Al-capping layer provides a local barrier for impurity incorporation by atmosphere exposure as chemical reactions or Ca with H_2_O and O_2_ are delayed [30,31].

**Figure 3:**
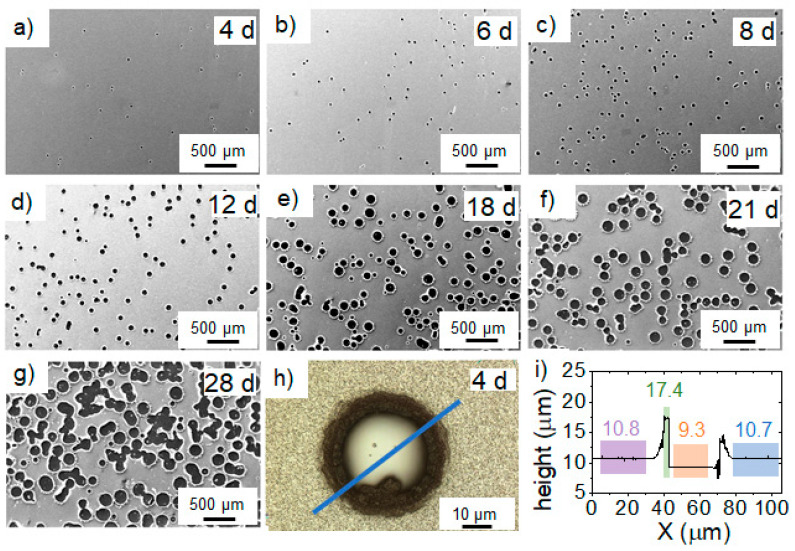
SEM images of Al-capped Ca deposited at 100 °C after exposure times (expressed in days (d)) of (**a**) 4 d, (**b**) 6 d, (**c**) 8 d, (**d**) 12 d, (**e**) 18 d, (**f**) 21 d, and (**g**) 28 d. (**h**) LOM image of a representative surface feature with 150X magnification after exposure time of 4 d. (**i**) LOM height profile across the surface feature in the capped Ca along the blue line depicted in h. The values indicate averaged heights of the marked sections according to the LOM coordinates in µm. The ridge height (at the crater edge) is around 8 μm with respect to the substrate (the middle part of the crater marked as orange).

The chemical composition data of Al-capped Ca was complemented by repeated SEM surface studies as well as LOM height-profiling measurements, see Figure 3.

For an exposure time of 4 days (Figure 3a), dark features are observed on capped Ca surface by SEM which increase in number and size with prolonged exposure time, see Figure 3a–g. The average feature diameter is approximately 30 µm after 4 days of atmosphere exposure, which increases to 200 µm after 38 days. Based on local chemical composition analysis by EDX, it is inferred that the features represent craters as the EDX signal is dominated by Si stemming from the substrate. EDX reveals a striking O enrichment of up to ~49 at % at the crater edge compared to the featureless surroundings where the O concentration was measured to be 3.1 ± 0.1 at %. Moreover, height profiling by LOM, depicted in Figure 3i, confirms the crater formation notion as the height difference of 1.4 µm between intact film and center of the crater is on the order of the film thickness. In addition, the edge of the crater exhibits a maximum height of approximately 8 µm above the substrate surface. As we seek to prevent impurity incorporation, this rather intriguing crater formation mechanism is not explored further.

It should be noted that the here reported O concentrations for the capped Ca determined by EDX and displayed in Figure 2, correspond to Ca film regions where the capping is intact. Based on these findings, it can be concluded that Al-capping provides temporally limited local protection of elemental Ca (see Section 3.2.1 for further information).

### 3.2. Impurity Incorporation under Atmosphere Exposure 

#### 3.2.1. Atmosphere Exposure Time

**Figure 4:**
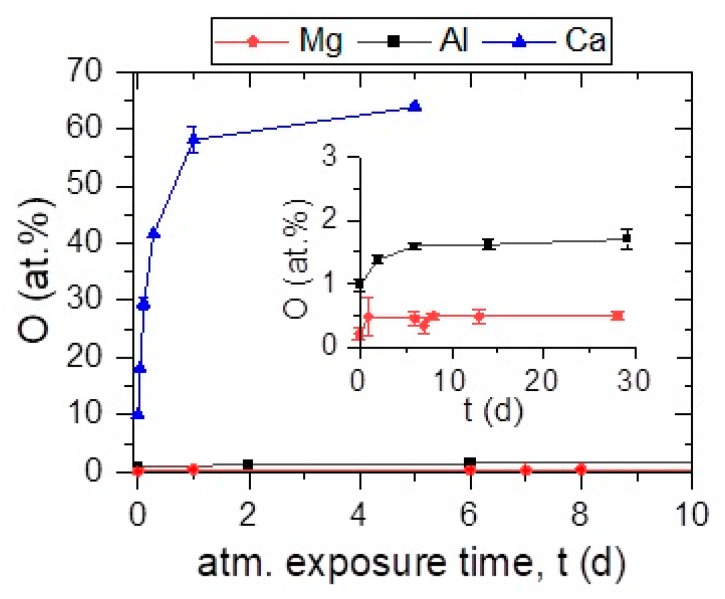
O concentration measured by EDX in Mg (base pressure = 6.1 × 10^−6^ Pa), Al (base pressure = 8.2 × 10^−6^ Pa), and Ca (base pressure = 6.5 × 10^−6^ Pa) thin films synthesized at 100 °C as a function of their atmosphere exposure time after synthesis (expressed in days (d)). Atmosphere exposure time of 0 days corresponds to the as-deposited state (≤3 min atmosphere exposure). Thin film delamination was observed in case of Ca after 5 days preventing further analysis.

Here, Mg, Al, and Ca were synthesized at 100 °C and subsequently exposed to atmosphere without capping. O incorporation has been monitored by measuring the O concentration repeatedly by EDX after atmosphere exposure of up to 29 days, see Figure 4. The inset shows impurity concentrations in Mg and Al thin films for longer exposure times.

As discussed before, in the as-deposited state, which corresponds to an air exposure time of ≤3 min required by the transport of the sample from the deposition chamber to the measurement device, Ca exhibited an O concentration of 10.0 ± 0.4 at % while a concentration of 0.2 ± 0.1 and 1.0 ± 0.1 at % was measured for Mg, and Al, respectively.

For Mg, no change is observed in O uptake within the measured time span of 29 days under atmosphere exposure at RT. Thus, Mg films are considered to be stable which can be explained by the formation of a thin MgO layer on the surface that despite a Pilling– Bedworth ratio of 0.8 is protective at temperatures below 450 °C preventing further oxidation [32,33].

For Al, the measured O concentration increases from 1.0 ± 0.1 to 1.6 ± 0.1 at % within 6 days of atmosphere exposure and a steady state is reached afterwards. The low O uptake of Al is attributed to the formation of Al_2_O_3_ on the surface which protects the Al thin film from further oxidation [34]. The initial increase of O uptake could be attributed to the time-dependent oxide growth. By reaching a critical oxide thickness, film growth ceases [35]. Furthermore, Al has a Pilling–Bedworth ratio of 1.3 indicating that Al_2_O_3_ is a protective oxide [36].

In contrast, elemental Ca shows a continuous O uptake upon atmosphere exposure as described in Section 3.1. To investigate topographic changes during Ca thin film oxidation, repeated SEM analyses were performed (see Supplementary Materials Figure S1). In the as-deposited state, bright spots are observed on the Ca surface which grow in number and size when the film is exposed to atmosphere. Local chemical composition analysis by EDX revealed an O enrichment at the bright spots compared to the Ca-rich surrounding. The film is covered with these spots after 24 h (Figure S1e). Extensive film delamination is observed after 5 days (Figure S1f). 

**Figure 5:**
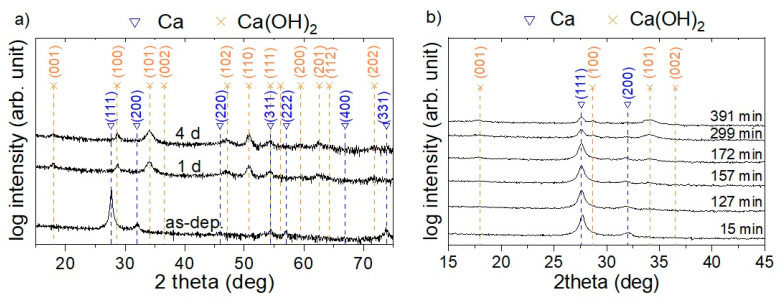
Diffractograms of Ca thin film deposited at 100 °C in (**a**) as-deposited state (≤3 min atmosphere exposure) and after 1 and 4 days of atmosphere exposure obtained using Bragg–Brentano (2° offset) scans and (**b**) for varying atmosphere exposure times using a large area detector.

Other than chemical composition and topographic changes of Ca thin film for varying atmosphere exposure times, structural modifications were monitored by XRD (Figure 5).

In the as-deposited state with ≤3 min atmosphere exposure, the obtained peaks can be attributed to the formation of the cubic Ca phase (JCPDS card 23-0430) indicating XRD phase pure Ca formation (Figure 5a). In contrast, after one day of atmosphere exposure, the Ca peaks vanish while newly emerging peaks can all be attributed to Ca(OH)_2_ (JCPDS card 44-1481) indicating a complete transformation of the Ca thin film to this phase within one day, which is in agreement with the observed surface modifications using SEM (see Figure S1). Furthermore, a measured O concentration of around 60 at % by EDX after 1 day of atmosphere exposure, displayed in Figure 2 and Figure 4, agrees reasonably well with the formation of Ca(OH)_2_ considering that H cannot be detected by EDX [37]. No structural changes between 1 and 4 days of exposure time are observed by XRD, Figure 5a.

To further investigate the transition from Ca to Ca(OH)_2_, large area detector XRD scans were performed for atmosphere exposure times of 15 up to 400 min, see Figure 5b. After an exposure time of around 170 min, the formation of crystalline Ca(OH)_2_ is observed. Ca peak intensities decrease continuously over time underlining a continuous transformation of elemental Ca to Ca(OH)_2_.

Based on these findings, it may be speculated that the reason for the formation of initial bright spots (see Figure S1) could be the presence of surface defects causing H_2_O adsorption which then provides initial nucleation sites for formation of Ca(OH)_2_. This is in agreement with the fact that Ca reacts with water vapor over the temperature range of 20–150 °C [30].

In summary, it has been demonstrated that Ca is not stable in atmosphere, transforming into Ca(OH)_2_ within one day, while Al exhibits a continuous increase of O within the first days in air until a steady state is reached. For Mg, no O incorporation upon atmosphere exposure was observed. Hence, Mg and Al form self-passivating surface layers upon atmosphere exposure.

#### 3.2.2. Influence of Synthesis Temperature

Mg and Al thin films were synthesized at different temperatures. Since Ca films are unstable upon atmosphere exposure, the influence of synthesis temperature could not be studied for this material.

**Figure 6:**
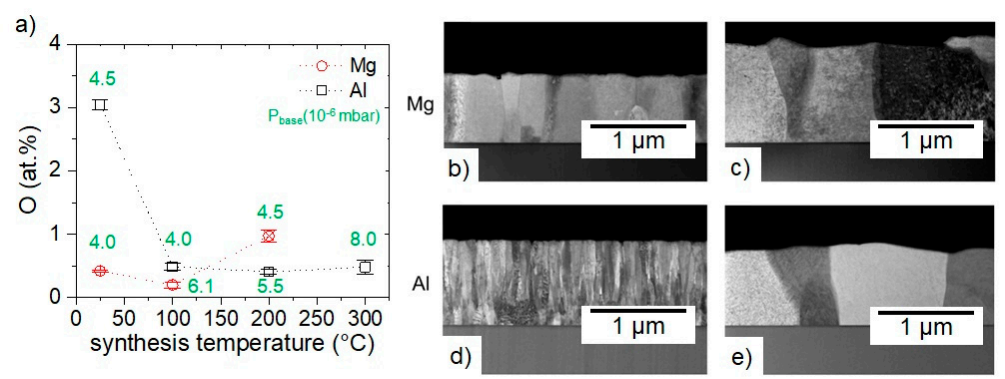
(**a**) O concentrations measured by EDX as a function of synthesis temperature for Mg and Al films in the as-deposited state (minimized air exposure time of ≤3 min). The red x marks the synthesis temperature where partial desorption of Mg is observed. The green values indicate the base pressure (P_base_) at synthesis temperature before deposition. STEM images of Mg synthesized at (**b**) RT and (**c**) 100 °C; Al synthesized at (**d**) RT and (**e**) 200 °C.

EDX measured as-deposited O concentrations with ≤3 min atmosphere exposure as a function of synthesis temperature of the films are displayed in Figure 6a. To investigate the mechanism of O incorporation, STEM was performed on the cross-section of the films deposited at RT and one film synthesized at a higher temperature for each material, see Figure 6b–e.

Mg films synthesized at RT and at 100 °C show similar O concentrations of 0.4 ± 0.1, 0.2 ± 0.1, respectively whereas the film deposited at 200 °C exhibits an O concentration of 1.0 ± 0.1 at % (Figure 6a). It should be noted that the Mg film synthesized at 200 °C has a 60% lower thickness than the other films suggesting the onset of desorption of Mg which was also reported for Mg in Mg-Ca-Al thin film occurring due to the high Mg vapor pressure [38,39].

Comparable O incorporations observed in the Mg films synthesized at RT and 100 °C can be explained by their microstructure. Mg films synthesized at RT show a dense columnar microstructure with grain boundaries almost perpendicular to the substrate, see Figure 6b. Furthermore, Mg films synthesized at 100 °C show a columnar microstructure similar to that of the film synthesized at RT, see Figure 6c. However, the film synthesized at 100 °C exhibits larger grains which is attributed to the higher substrate temperature leading to larger adatom mobility and activated grain boundary mobility causing larger grain sizes [21]. The absence of a difference in the measured O concentration is, hence, explained by the comparable, dense microstructure in both Mg films potentially preventing severe O uptake upon atmosphere exposure. Furthermore, based on these findings, an upper limit in synthesis temperature of highly pure Mg is obtained to be between 100 and 200 °C, Figure 6a.

Al films synthesized at RT exhibit an O concentration of 3.0 ± 0.1 at % while the films synthesized at 100, 200, and 300 °C show a lower and almost constant O concentration of 0.5 ± 0.1, 0.4 ± 0.1 and 0.5 ± 0.1 at %, respectively.

The high O concentration in RT synthesized Al film is attributed to the under-dense columnar microstructure observed in STEM, displayed in Figure 6d, which can serve as diffusion paths for O incorporation [40]. Consequently, high O concentration could be a result of O incorporation induced by atmosphere exposure occurring during the transfer of the film to the measurement device despite the short transportation time of ≤3 min. In contrast to the RT synthesized film, Al film synthesized at 200 °C depicted in Figure 6e, shows a columnar microstructure similar to that of Mg film synthesized at 100 °C. Hence, upon densification of the microstructure in Al thin films by increasing the synthesis temperature from RT to ≥100 °C, the O uptake by atmosphere exposure can be reduced significantly, see Figure 6a.

Based on the Mg (Al) melting point at 650.0 °C (660.5 °C) [41], homologous temperatures of the film synthesized at RT (=25 °C) and 100 °C are calculated to be 0.32 (0.32) and 0.40 (0.40), respectively. Movchan and Demchishin [20] published a homologous temperature of 0.3 as boundary between zone I and II where the latter morphology was attributed to the initiation of surface diffusion, while the transition between zone II and III, occurring at a homologous temperature of 0.5, is attributed to activation of bulk diffusion. Considering that the metals primarily investigated by Movchan and Demchishin were Ti, Ni, and W and the film thicknesses were in the range of 0.3 to 2 mm, the magnitude of the boundary values of 0.3 (zone I → II) and 0.5 (zone II → III) may or may not be relevant for the rather low melting point materials investigated here. Petrov et al. noted that the boundaries between the zones are diffuse and ‘‘transitions’’ occur gradually over wide ranges in homologous temperature [11]. Furthermore, Sanders [42] and Barna [43] proposed revised zone boundary values: a homologous temperature of 0.1 marks the boundary between zone I and T where the latter morphology was attributed to the initiation of surface diffusion, whereas a homologous temperature of 0.3 was determined as the transition between zone T and II. Here, in addition to surface diffusion also bulk diffusion is activated and grain boundary mobility is observed. Depla et al. [44] demonstrated that these boundaries quantitatively affect several film properties. The cross-sectional images of all here reported Mg films indicate that surface and bulk diffusion processes were active, whereas for Al this is only observed at a homologous temperature of 0.4 but not for 0.3. Furthermore, the homologous temperature of the Mg and Al films synthesized at 200 °C is calculated to be 0.51. At this temperature, evidence for Mg desorption has been obtained, indicating that the morphology evolution is, in addition to surface and bulk diffusion, also affected by Mg desorption. Futher, for Al deposited at 300 °C the homologous temperature is 0.61 suggesting that the morphology is strongly affected by bulk diffusion and grain boundary mobility. 

## 4. Conclusions

The goal of this study was to contribute towards a better understanding of the impurity incorporation after deposition by atmosphere exposure in Mg, Al, and Ca thin films synthesized by magnetron sputtering to subsequently be able to improve film purity.

It is evident that Mg and Al form native self-passivating layers upon atmosphere exposure rendering them as suitable capping layer materials. However, Al-capped Ca thin films are only locally protected and are unstable upon atmosphere exposure.

While the morphology evolution for both, Mg and Al thin films are dependent on the substrate temperature, mediated by surface diffusion, bulk diffusion, grain boundary motion, and evaporation. The film morphologies exhibiting the lowest oxygen concentrations, based on EDX, are observed at a homologous temperature of 0.4. Furthermore, all Mg and Al films form native self-passivation layers. The efficiency thereof in terms of hindering further oxidation of the films in atmosphere is strongly dependent on the underlying morphology, which in turn is defined by the growth temperature.

Based on these findings, it can be learned that the film morphology as well as the ability to form native passivation layers govern the magnitude and temporal evolution of the atmosphere exposure induced impurity incorporation.

## Figures and Tables

**Table 1:** Synthesis parameters of thin films deposited within this study. Room temperature is denoted as RT. The Ar partial pressure was kept constant for all depositions at 0.5 Pa.

Film	Substrate Temperature (°C)	Base Pressure (10^−6^ Pa)	DC Target Power (W)	Al-Capping
Mg	RT	4.0	50	×
	100	6.1	50	×
	100	7.0	50	✓
	200	4.5	50	×
Al	RT	4.5	200	×
	100	4.0	200	×
	100	8.2	200	×
	200	5.5	200	×
	300	8.0	200	×
Ca	100	5.0	65	✓
	100	6.5	100	×

## Data Availability

The authors declare that all relevant data supporting the findings of this study are available within the article and its supplementary information.

## Supplementary Materials

The following supporting information can be downloaded at: https://www.mdpi.com/article/10.3390/ma16010414/s1, Figure S1: SEM images of Ca thin film surface in (a) as-deposited state as well as after atmosphere exposure times of (b) 50 min, (c) 140 min, (d) 400 min, (e) 24 h, (f) 5 d.
