# Supplementary material for: Effect of Growth Temperature and Atmosphere Exposure Time on Impurity Incorporation in Sputtered Mg, Al, and Ca Thin Films
Source: Materials (Basel). 2023 Jan 1;16(1):414. doi: 10.3390/ma16010414 (PMC9822154; doi:10.3390/ma16010414)
Supplement: Supplementary file 1 [file materials-16-00414-s001.zip › materials-2050249-supplementary.pdf]

materials

MDPI

Article

Effect of Growth Temperature and Atmosphere Exposure Time on Impurity Incorporation in Sputtered Mg, Al, and Ca Thin Films

Shamsa Aliramaji [1,*], Philipp Keuter [1], Deborah Neuß [1], Marcus Hans [1], Daniel Primetzhofer [2], Diederik Depla [3] and Jochen M. Schneider [1]

1 Materials Chemistry, RWTH Aachen University, Kopernikusstr. 10, D-52074 Aachen, Germany
2 Department of Physics and Astronomy, Uppsala University, Box 516, S-75120 Uppsala, Sweden
3 Department of Solid State Sciences, Ghent University, Krijgslaan 281 (S1), B-9000 Gent, Belgium
* Correspondence: aliramaji@mch.rwth-aachen.de

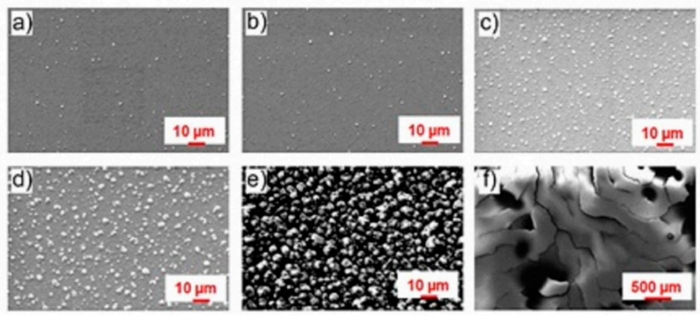

Figure S1. SEM images of Ca thin film surface in (a) as-deposited state as well as after atmosphere exposure times of (b) 50 min, (c) 140 min, (d) 400 min, (e) 24 h, (f) 5 d.
